# DHA Supplementation Alone or in Combination with Other Nutrients Does not Modulate Cerebral Hemodynamics or Cognitive Function in Healthy Older Adults

**DOI:** 10.3390/nu8020086

**Published:** 2016-02-09

**Authors:** Philippa A. Jackson, Joanne S. Forster, J. Gordon Bell, James R. Dick, Irene Younger, David O. Kennedy

**Affiliations:** 1 Brain, Performance and Nutrition Research Centre, Northumbria University, Newcastle-upon-Tyne NE1 8ST, UK; jo.forster@northumbria.ac.uk (J.S.F.); david.kennedy@northumbria.ac.uk (D.O.K.); 2 Institute of Aquaculture, University of Stirling, Stirlingshire FK9 4LA, UK; g.j.bell@stir.ac.uk (J.G.B.); j.r.dick@stir.ac.uk (J.R.D.); Irene.younger@stir.ac.uk (I.Y.)

**Keywords:** DHA, EPA, omega-3 polyunsaturated fatty acids, NIRS, cerebral blood flow, cognitive decline

## Abstract

A number of recent trials have demonstrated positive effects of dietary supplementation with the omega-3 polyunsaturated fatty acids (*n*-3 PUFAs), docosahexaenoic acid (DHA) and eicosapentaenoic acid (EPA) on measures of cognitive function in healthy young and older adults. One potential mechanism by which EPA, and DHA in particular, may exert these effects is via modulation of cerebral hemodynamics. In order to investigate the effects of DHA alone or provided as one component of a multinutrient supplement (also including Gingko biloba, phosphatidylserine and vitamins B_9_ and B_12_) on measures of cerebral hemodynamics and cognitive function, 86 healthy older adults aged 50–70 years who reported subjective memory deficits were recruited to take part in a six month daily dietary supplementation trial. Relative changes in the concentration of oxygenated hemoglobin and deoxygenated hemoglobin were assessed using Near Infrared Spectroscopy (NIRS) during the performance of cognitive tasks prior to and following the intervention period. Performance on the cognitive tasks was also assessed. No effect of either active treatment was found for any of the NIRS measures or on the cognitive performance tasks, although the study was limited by a number of factors. Further work should continue to evaluate more holistic approaches to cognitive aging.

## 1. Introduction

Declining cognitive function is associated with increasing age. In order to combat the global health burden of cognitive decline associated with societal ageing, modifiable lifestyle factors known to attenuate cognitive ageing such as exercise [1] and diet [2,3] are increasingly being explored as preventative approaches. A number of large-scale epidemiological studies indicate that intake of one potentially protective group of dietary components, omega-3 polyunsaturated fatty acids (*n*-3 PUFAs), is inversely associated with reduced risk of cognitive decline and dementia [2,4,5,6,7,8], although this finding is not universally reported [9]. Indeed, although, as a whole, the results from randomized controlled trials which have assessed the effects of supplemental *n*-3 PUFAs on cognitive function in older adults have yielded mixed results, more recent trials that have administered the *n*-3 PUFA docosahexaenoic acid (DHA) in dosages in excess of 850 mg/day for at least 6 months have reported more consistent positive effects of treatment on cognitive performance outcomes [10,11,12,13,14]. 

The mechanisms underpinning these effects in humans remain unclear but are likely multi-factorial. For example, evidence from *in vitro* and *in vivo* studies has revealed a role for DHA in promoting synaptic plasticity [15,16,17,18] and neurogenesis [19,20]. DHA has also been shown to have a number of neuroprotective effects including suppressing pro-inflammatory pathways and upregulating pro-resolving mediators such as neuroprotectin D1, modulating mitochondrial function and reducing oxidative stress [21]. In humans, one recent trial revealed that 2.2 g/day *n*-3 PUFAs for 26 weeks in healthy, cognitively intact older adults resulted in improved performance on executive function tasks [12]. This finding was associated with improved brain white matter microstructural integrity and gray matter volume measured using fMRI, suggesting the observed effects of *n*-3 PUFAs on cognition may be related to their upregulating effect on myelination [22], neurogenesis [19,23] and synaptogenesis [24]. 

Dietary DHA is also know to modulate a number of neurotransmitter systems including the cholinergic system [25], which is known to play a key role in memory and learning [26] and the degradation of which is associated with the typical cognitive deficits observed in normal and pathological aging [27,28]. One group of researchers also demonstrated a role for cholinergic neurotransmission in the regulation of the neurovascular coupling of brain activity and local cerebral blood flow in aged monkeys [29], and subsequently demonstrated that the age-impaired regional cerebral blood flow (rCBF) response to tactile stimulation was positively modulated following four weeks’ dietary supplementation with 150 mg/kg/day DHA [30]. These studies suggest that DHA facilitates neurovascular coupling via modulation of the cholinergic system and that improved regulation of CBF may be another mechanism through which DHA is associated with enhanced cognitive function. In support of this, using near infrared spectroscopy (NIRS), CBF effects of DHA-rich fish oil were demonstrated in healthy young adults [31]. This study revealed a pattern of increased CBF in the pre-frontal cortex during completion of cognitive tasks following daily treatment with 1 g or 2 g DHA-rich fish oil for 12 weeks, compared to placebo. However, this study failed to show any effects of either dose on cognition. This may be due in part to the fact that the observed effects on CBF—and therefore the increased delivery of metabolic substrates to active tissue—were too mild to result in enhanced performance in a sample of healthy adults (18–35 years) already at their cognitive peak.

It is also possible that *n*-3 PUFAs, in combination with other supporting compounds relevant to brain function, may be more effective than treatment with *n*-3 PUFAs in isolation. Indeed, *n*-3 PUFA multinutrient approaches to treating patients with mild to moderate Alzheimer’s disease [32,33] have yielded promising preliminary results with regard to improved cognitive function. In addition, a recent pilot study reported by Strike *et al*. [14] revealed that a DHA-rich fish oil multinutrient supplement also containing phosphatidylserine (PS), vitamin B_12_, folic acid and *Ginkgo biloba* resulted in improved working memory performance and measures of mobility in healthy non-demented older females. However, the study may have been underpowered to compare the effects of the active treatment against placebo. The current study therefore aimed to evaluate the effects of six months’ supplementation with the same multinutrient, compared with placebo, on cerebral hemodynamics and cognitive function in a sample of older adults aged 50–70 years. In order to evaluate the individual contribution of DHA contained within the multinutrient supplement, the effect of DHA-rich fish oil containing the same amount of *n*-3 PUFAs as the multinutrient supplement was also investigated.

## 2. Experimental Section

Data for this double-blind, randomized, placebo-controlled parallel groups study was collected between July 2010 and June 2012. All testing took place within the Brain, Performance and Nutrition Research Centre at Northumbria University in Newcastle upon Tyne, UK. The study received ethical approval from the Northumbria University School of Psychology and Sport Sciences Ethics Committee and was conducted according to the Declaration of Helsinki (1964). All participants gave their informed consent before their inclusion in the study. The trial (NCT01185379) is registered at www.clinicaltrials.gov.

### 2.1. Participants

A total of 248 healthy adults who all reported subjective memory deficits in the absence of cognitive impairment (MMSE scores ≥ 26) were recruited from the general public by means of newspaper and radio advertisements and targeted mailshots in the Newcastle upon Tyne area. All participants also reported themselves to be healthy, had a BMI ≤ 35, were normotensive (blood pressure ≤ 139/89), smoked less than 15 cigarettes per day, had no history of alcohol or drug abuse, did not have a current diagnosis of anxiety or depression or a history of neurological or psychiatric illness, were not taking any blood-thinning, cholesterol-lowering or antidepressant medications, were not taking any nutritional supplements containing the same ingredients as the experimental treatments and consumed no more than one portion of oily fish per month. 

### 2.2. Study Design

**Figure 1:**
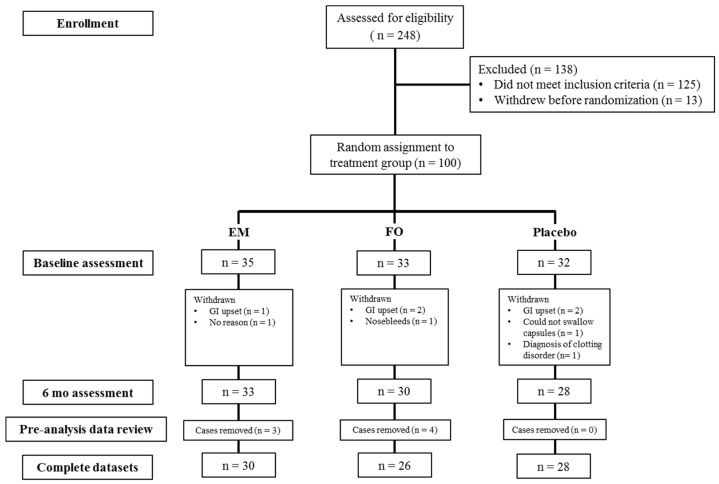
Consort diagram showing the flow of subjects at each stage of data collection and analysis. EM, docosahexaenoic acid (DHA)-rich fish oil multinutrient; FO, DHA-rich fish oil; GI, gastrointestinal; mo, months.

A randomized, placebo-controlled, double-blind, parallel groups design was used (Figure 1). Eligible volunteers were randomized to one of three treatment groups according to a computer-generated randomization schedule (www.randomization.com). The three treatment groups were: 2 g DHA-rich fish oil (896 mg DHA, 128 mg EPA), a multinutrient (Efalex Active 50+) containing 2 g DHA-rich fish oil (946.4 mg DHA, 160 mg EPA) with added phosphatidylserine (PS, 88 mg), *Ginkgo biloba* (240 mg), folic acid (1 mg) and vitamin B_12_ (24 mg) or placebo. All capsules were provided by Efamol Ltd., UK. Participants were instructed to consume 4 × 500 mg capsules daily for 6 months which were divided between breakfast and their evening meal to avoid gastrointestinal upset. The dosage of fish oil was selected on the basis that it equates to a level of DHA intake achievable by dietary sources alone (2–3 oily fish meals/week) and also based on previous studies that have demonstrated this dose to be effective for modulating cognition and cerebral blood flow in healthy young adults [31]. The placebo capsules contained 2.24 g high oleic acid sunflower oil and 120 mg fish oil (32 mg DHA + EPA) for masking purposes. All capsules were packaged, labelled and randomized on-site by a third party who had no further involvement in the study. In addition, due to the fact that the color of the DHA-rich fish oil capsules did not match the other two treatments, all treatments were collected and counted by a third party who had no further involvement in the study.

#### 2.2.1. Near Infrared Spectroscopy (NIRS)

Functional near infrared spectroscopy (NIRS) is a non-invasive neuroimaging technique that is used to monitor tissue oxygen status. When applied to the intact skull, relative changes in the concentration of oxygenated (oxy-Hb) and deoxygenated (deoxy-Hb) hemoglobin in the superficial cortex can be used to measure CBF during localized neural activity. The application of NIRS in nutritional neuroscience is gaining momentum, with a growing number of studies demonstrating the sensitivity of NIRS to changes in cerebral hemodynamic response to cognitive tasks following acute administration of polyphenols [34,35,36,37] and caffeine [38] and also following 12 weeks’ supplementation with omega-3 fatty acids in younger and older healthy adults [31,39]. 

In the present study, relative changes in the absorption of near infrared light were measured using a 2-channel continuous-wave Oxymon system (Artinis Medical Systems B.V., Zetten, The Netherlands). Two wavelengths of light (approximately 765 and 855 nm) were introduced through the skull via a laser emitter and measured, following transit through the upper surface of the cortex, by an optode placed 40 mm from the light source. Optodes were placed on either side of the forehead in positions corresponding to Fp1 and Fp2 of the International 10–20 system and held in place with an optode holder and headband. Relative concentration changes in oxygenated hemoglobin (Hb), deoxygenated Hb, and total Hb were calculated by means of a modified Beer-Lambert law within the proprietary software [40]. NIRS data were time stamped by the researcher at the start and end of each task so that only NIRS data collected during the tasks were analyzed.

Given that continuous-wave NIRS generates concentration change data that is intrinsically baseline-adjusted to the concentration immediately prior to the first data point in the recording session, it cannot be used to quantify gross changes in CBF parameters that take place between two separate recording sessions. In this instance the change from baseline data generated by the NIRS system was subjected to a second baseline adjustment by creating ‘change from baseline’ data with respect to the 10 min of NIRS data collected immediately prior to completing the tasks—this therefore provided a more accurate baseline measure of immediately pre-task NIRS parameters.

#### 2.2.2. Cognitive Tasks

The tasks selected for use in this study have previously been shown to activate the frontal cortex using NIRS [34,37,38] and have been described in full elsewhere [34]. Briefly, participants completed four repetitions of the ‘Cognitive Demand Battery’ (CDB), a 9-min collection of three tasks. The CDB comprised two minutes each of Serial 3 and Serial 7 subtractions wherein the participant had to keep subtracting 3 (or 7) from a random starting number between 800 and 999 as fast and as accurately as they could for the duration of the task. Each three digit response made by using the linear computer number keys was represented on-screen by an asterisk, so participants had to keep track of the number they last entered in order to gain the best possible score. Performance data (total number of subtractions and number of errors) were calculated for the Serial 3 s and 7 s elements separately. The serial subtraction tasks were followed by the Rapid Visual Information Processing (RVIP) task, a 5-min sustained attention task wherein participants monitored a series of rapidly presented (100/min) digits for sequences of three odd or even numbers in a row. Eight correct target strings were presented each minute and performance was measured as accuracy of correctly identified target strings and average reaction time for correct detections. The battery was concluded with participants rating how mentally fatigued they felt at that moment in time on a visual analogue scale with the 100 mm line being anchored by ‘not at all’ and ‘extremely’ at either end. All the tasks were presented using the COMPASS cognitive assessment system (Northumbria University, Newcastle upon Tyne, UK).

#### 2.2.3. Blood Sample Collection and Preparation

Fingertip whole blood was collected using a blood lance (Accu-Chek, Safe-T-Pro Plus, Roche Diagnostics GmbH, Mannheim, Germany). Blood samples were collected by dropping a spot of blood straight from the fingertip onto pre-prepared Whatman 903 cards saturated with butylated hydroxytoluene (BHT; 50 mg/100 mL in ethanol) on each collection spot. Four blood spots were collected in total at each sampling point. Sampling cards were air-dried for 3 h before being stored frozen in individual Zip lock bags with desiccant at −80 °C until analysis.

The dried whole blood sample was detached from the collection device using forceps and placed into a screw-cap vial containing 1 mL of a methylating solution (1.25 M-methanol–HCl). The vials were placed in a hot block at 70 °C for 1 h. The vials were allowed to cool to room temperature and then 2 mL of distilled water and 2 mL of saturated HCl solution were added. Fatty acid methyl esters (FAME) were then extracted using 2 × 2 mL of isohexane and purified by solid phase extraction (UCT, 500mg Silica, Bartram Road, Bristol, PA, USA).

#### 2.2.4. Whole Blood Fatty Acid Analysis

FAME were separated and quantified by GLC (ThermoFisher Trace, Hemel Hempstead, Hertfordshire, UK) using a 60 m × 0.32 mm i.d. × 0.25 µm film thickness capillary column (ZB Wax; Phenomenex, Macclesfield, Cheshire, UK). H_2_ was used as a carrier gas at a flow rate of 4.0 mL/min and the temperature program was from 50 to 150 °C at 40 °C/min then to 195 °C at 2 °C/min and finally to 215 °C at 0.5 °C/min. Individual FAME were identified compared to well-characterized in-house standards as well as commercial FAME mixtures (Supelco 37 FAME mix; Sigma-Aldrich Limited, Gillingham, Dorset, UK). In contrast with venous blood sampling, this validated method of fatty acid analysis has the advantage of being non-invasive, rapid and comparatively inexpensive [41].

### 2.3. Procedure

Participants attended the laboratory on four separate occasions. At the first introductory visit participants completed informed consent procedures and were screened according to the inclusion/exclusion criteria. General demographic and anthropomorphic data including age, number of years of formal education and height and weight were collected. Participants were then trained on the cognitive tasks. In total, three practice trials for each task were completed to reduce the possibility of practice effects at baseline. The second visit comprised the baseline NIRS assessment which was completed within 28 days of the introductory visit and took place between 2 and 5 pm. Participants were instructed to consume nothing but water one hour prior to the assessment. All food and drinks that they had consumed that day prior to the session were recorded. Participants were then fitted with the NIRS headband and completed a 10-minute rest period whilst watching a non-stimulating lifestyle programme on a laptop. Following this, participants completed one repetition of the CDB (10 min). Participants were then provided with a supply of three months’ of capsules in an opaque container. They were instructed to refrain from consuming oily fish and dietary supplements containing *n*-3 PUFAs for the duration of the six month intervention. After three months, participants attended the laboratory for a third visit to receive a second batch of capsules. Adverse events were monitored but no other study procedures took place at this visit. The fourth and final visit comprised a final NIRS assessment, again taking place in the afternoon between 2 and 5 pm. Participants were sent a reminder for the final assessment which included details of what they had to eat and drink prior to the baseline assessment and were instructed to follow the same diet prior to the assessment. Following adverse event monitoring, participants were again fitted with the NIRS headband and completed a 10-minute rest period whilst watching a non-stimulating lifestyle programme on a laptop. Following this, participants completed four repetitions of the CDB (40 min). 

### 2.4. Statistical Analysis

Calculation of an appropriate sample size was made utilizing treatment effect sizes for both the cerebral blood flow and cognitive task elements of the study derived from a previous study that assessed the effects of DHA-rich oil on cognition and cerebral blood flow parameters in healthy young adults [31]. Assuming similar effect sizes (*f* = 0.35 and *f* = 0.26 for CBF and CDB, respectively), the total sample size required in order to achieve 80% power at an alpha level of 0.05 was 48 for the NIRS outcomes and 90 for the CDB outcomes. The sample size was therefore set at 99 participants to account for a 10% dropout. Power calculations were made using GPower 3.1.1. (Faul, Erdfelder *et al.* [42]).

The primary analysis of the NIRS total-Hb data was conducted by repeated measures ANOVA including treatment (Placebo, FO, EM) as a between subjects term and task (12 levels; 4 repetitions of each of the three timed CDB tasks) as a within subjects term. Baseline percentage of whole blood DHA concentration, number of years in formal education and age were included in the model as covariates.

The primary analysis of the cognitive performance and mental fatigue data was also by repeated measures ANOVA including treatment (Placebo, FO, EM) as a between subjects term and repetition (1–4) as a within subjects term. Baseline whole blood DHA concentration, number of years in formal education, age and respective pre-dose score for each task outcome were included in the model as covariates. 

## 3. Results

### 3.1. Baseline Differences

Between group differences on the demographic and whole blood fatty acid status variables at baseline were analyzed by one-way ANOVA and chi-squared tests, where appropriate. See Table 1.

**Table 1:** Baseline participant characteristics. EM, DHA-rich fish oil multinutrient; FO, DHA-rich fish oil.

	EM (*n* = 30)	FO (*n* = 26)	Placebo (*n* = 28)	*p* ^1^
Male/Female	12/18	10/16	10/18	0.503
Age (years)	59.87 ± 4.70 ^2^	60.31 ± 4.89	59.64 ± 5.28	0.883
BMI (kg/m^2^)	26.17 ± 3.44	25.65 ± 3.29	25.76 ± 4.25	0.857
Blood pressure (systolic mmHg)	120 ± 11	123 ± 14	124 ± 15	0.437
Blood pressure (diastolic mmHg)	81 ± 8	83 ± 8	84 ± 7	0.293
Handedness (left/right)	3/27	2/24	2/26	0.471
Years in education	13.30 ± 3.16	13.54 ± 3.39	14.86 ± 3.95	0.205

^1^ Values were derived from one-way ANOVA or chi-squared test (2-tailed), where appropriate.

^2^ Means and standard deviations are presented, where appropriate.

**Figure 2:**
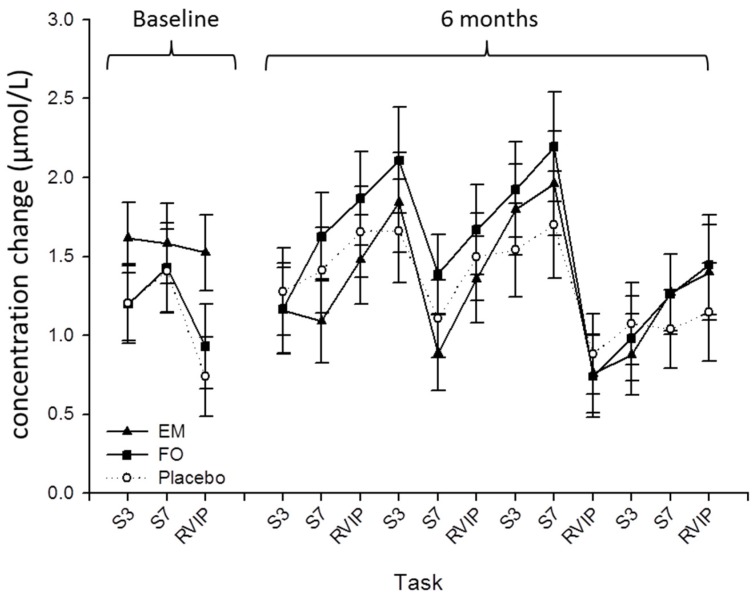
Cerebral blood flow as indexed by concentration changes in total levels of hemoglobin in the prefrontal cortex during completion of one repetition of the Cognitive Demand Battery (CDB) at Baseline, and four repetitions of the CDB after taking the supplement for 6 months. Data points are derived from averages of the task periods, with SEM error bars and are adjusted for age, years in education, baseline whole blood DHA. EM, DHA-rich fish oil multinutrient; FO, DHA-rich fish oil; S3, Serial 3 subtractions; S7, Serial 7 subtractions; RVIP, Rapid Visual Information Processing.

Prior to the main analysis, group differences on performance on the cognitive tasks at the Baseline visit were explored with independent one-way ANOVAs for each of the task outcomes (Table 2). No significant effects of treatment group were observed. Similarly, repeated measures ANOVA of the Baseline visit cerebral blood flow parameters also showed no difference between groups (Figure 2).

**Table 2:** Mean scores and standard error for the Cognitive Demand Battery (CDB) task outcomes at Baseline and 6 months. Data for the 6 months assessment are adjusted for age, years in education, baseline whole blood DHA and baseline score. *F* values and probabilities derived from the repeated measures ANOVA are also presented. Rep, repetition of the CDB; EM, DHA-rich fish oil multinutrient; FO, DHA-rich fish oil; RVIP, Rapid Visual Information Processing; RT, reaction time.

		Baseline		Rep 1		Rep 2		Rep 3		Rep 4			*F*	*p*
		Mean	SE	Mean	SE	Mean	SE	Mean	SE	Mean	SE			
Serial 3 s total	EM	26.67	1.81	28.51	0.75	32.53	0.75	32.35	0.92	32.03	0.86	Treat	0.04	0.96
	FO	29.19	2.73	29.69	0.84	31.60	0.84	30.93	1.03	32.43	0.97	Treat × rep	1.21	0.31
	Placebo	28.54	2.24	28.53	0.79	31.70	0.80	31.60	0.98	32.47	0.91			
Serial 3 s error	EM	2.27	0.42	2.19	0.45	1.93	0.33	2.21	0.38	2.46	0.35	Treat	0.79	0.46
	FO	2.73	0.59	2.14	0.52	2.31	0.37	1.88	0.43	2.24	0.39	Treat × rep	0.41	0.87
	Placebo	1.86	0.26	2.88	0.48	2.36	0.35	2.65	0.41	2.42	0.37			
Serial 7 s total	EM	19.20	1.41	21.61	0.66	22.12	0.68	21.87	0.72	22.35	0.77	Treat	0.49	0.61
	FO	22.23	2.35	21.91	0.74	22.84	0.76	22.94	0.81	23.66	0.87	Treat × rep	0.58	0.727 ^1^
	Placebo	21.21	1.83	21.22	0.70	21.80	0.72	22.80	0.76	22.62	0.82			
Serial 7 s error	EM	2.80	0.67	3.36	0.56	2.76	0.39	2.37	0.33	2.73	0.41	Treat	0.19	0.83
	FO	3.12	0.47	2.73	0.62	2.92	0.44	2.53	0.37	3.10	0.46	Treat × rep	0.52	0.758 ^1^
	Placebo	2.93	0.35	2.65	0.59	3.05	0.41	2.15	0.35	2.47	0.44			
RVIP accuracy	EM	51.58	4.50	50.41	3.90	50.21	4.36	48.83	4.71	50.39	4.74	Treat	0.44	0.65
	FO	51.44	5.27	48.83	4.36	44.43	4.88	44.48	5.27	42.61	5.30	Treat × rep	0.54	0.760 ^1^
	Placebo	50.71	5.30	48.62	4.13	48.34	4.62	45.73	4.99	40.98	5.03			
RVIP RT	EM	497.62	10.07	492.84	20.09	504.63	28.75	472.02	31.67	495.97	32.43	Treat	1.43	0.25
	FO	473.45	21.44	462.49	22.42	431.84	32.10	437.02	35.35	404.81	36.20	Treat × rep	0.74	0.597 ^1^
	Placebo	477.06	19.60	498.49	21.33	465.37	30.53	436.43	33.62	434.72	34.43			
Mental fatigue	EM	54.03	2.99	54.57	3.20	59.44	3.60	65.94	3.71	71.35	3.52	Treat	0.60	0.55
	FO	58.15	4.24	57.06	3.59	66.98	4.03	72.21	4.16	76.32	3.95	Treat × rep	0.79	0.530 ^1^
	Placebo	51.89	4.48	53.39	3.40	63.71	3.82	70.33	3.94	74.89	3.74			

^1^ Greenhouse-Geisser adjusted degrees of freedom.

### 3.2. Compliance

Compliance to the daily supplementation regimen was assessed by capsule count at 3 and 6 months and verified with fingertip capillary whole blood measurements. Compliance was equally high in all three groups (EM: 90.1% ± 8.8%; FO: 91.3% ± 8.3%; Placebo: 91.0% ± 9.2%; *p* = 0.586). The high level of compliance was confirmed by analysis of the whole blood fatty acids (Table 3). Given that both active treatments provided similar quantities of *n*-3 PUFAs, it is not surprising that both treatments resulted in an increase of approximately 50% in whole blood DHA. Specifically, significant increases in the percentage of whole blood DHA, EPA and total *n*-3 PUFAs were observed at 6 months following both the EM and FO treatments, compared to placebo (all *p* < 0.001). Similarly, the percentage of arachidonic acid (AA) and total omega-6 PUFAs decreased in both the active treatment groups (all *p* < 0.001), as did the ratio of AA:EPA and AA:DHA (all *p* < 0.001).

**Table 3:** Capillary whole blood fatty acid concentrations and analysis. Mean percentage of total fatty acids ± SD are presented with F scores and probabilities derived from the ANCOVA of data collected at 6 months using the corresponding pre-dose Baseline value as the covariate. EM, DHA-rich fish oil multinutrient; FO, DHA-rich fish oil, AA, arachidonic acid; EPA, eicosapentaenoic acid; DHA, docosahexaenoic acid; *n*-6 PUFA omega-6 polyunsaturated fatty acids, *n*-3 PUFA, omega-3 polyunsaturated fatty acids.

		EM (*n* = 30)		FO (*n* = 26)		Placebo (*n* = 28)		*F*	*p*
		Mean	SD	Mean	SD	Mean	SD		
**AA**	Baseline	9.41	1.21	9.47	1.28	10.08	1.23	14.99	<0.001
	6 months	8.56 ^1^	1.08	8.56 ^1^	1.11	10.00 ^2,3^	1.16		
**Total *n*-6 PUFA**	Baseline	32.77	2.36	32.86	2.24	33.62	2.27	16.23	<0.001
	6 months	30.79 ^1^	2.34	31.32 ^1^	2.24	33.73 ^2,3^	2.07		
**EPA**	Baseline	1.08	0.45	0.93	0.28	1.02	0.36	19.81	<0.001
	6 months	1.69 ^1^	0.72	1.42 ^1^	0.49	0.98 ^2,3^	0.32		
**DHA**	Baseline	2.85	0.74	2.70	0.71	2.83	0.70	108.45	<0.001
	6 months	5.41 ^1^	1.11	5.56 ^1^	0.85	2.80 ^2,3^	0.63		
**Total *n*-3 PUFA**	Baseline	5.91	1.12	5.58	0.89	5.75	1.03	74.25	<0.001
	6 months	8.81 ^1^	1.77	8.56 ^1^	1.25	5.70 ^2,3^	1.00		
**AA:EPA**	Baseline	9.91	3.82	11.09	3.69	10.83	3.40	34.48	<0.001
	6 months	6.05 ^1^	3.07	6.60 ^1^	2.08	11.12 ^2,3^	3.54		
**AA:DHA**	Baseline	3.55	1.16	3.78	1.20	3.74	0.86	122.25	<0.001
	6 months	1.70 ^1^	0.68	1.59 ^1^	0.45	3.72 ^2,3^	0.80		

^1^ *p* < 0.001 derived from paired samples *t*-tests of data collected at Baseline and 6 months**.** ^2^ *p* < 0.001 derived from *post hoc* pairwise comparisons (Bonferroni) of Placebo and EM**.** ^3^ *p* < 0.001 derived from *post hoc* pairwise comparisons (Bonferroni) of Placebo and FO.

### 3.3. Near Infrared Spectroscopy

Raw NIRS data—*i.e*., relative concentration changes in each of the chromophores from an arbitrary time point (the time that the machine was switched on)—were converted to ‘change from baseline’ values where the baseline value was the average concentration change over the pre-task 10-min rest period. Change from baseline data were then averaged across each of the two minute (serial subtractions) or five minute (RVIP) task periods. In addition, since the primary aim of the study was to assess the effects of the interventions on global cerebral oxygenation during task performance, NIRS data from both of the hemispheres were combined.

Analysis of the NIRS data revealed no effect of treatment or interaction between treatment and task period for oxygenated, deoxygenated or total-Hb (Figure 2).

### 3.4. Cognitive Performance

No effect of treatment or interaction between treatment and repetition on any of the seven CDB task outcomes were observed (Table 2).

## 4. Discussion

The aim of the current study was to assess the effect of six months’ supplementation with a multinutrient dietary supplement containing a number of potentially cognitive enhancing components including DHA, phosphatidylserine (PS), vitamin B_12_, folic acid and *Ginkgo biloba* on cerebral hemodynamics and cognitive function in healthy older adults aged 50–70 years reporting subjective memory complaints. The separate effects of DHA-rich FO were also compared against placebo. The capillary whole blood fatty acid analyses revealed a doubling in concentration of DHA and around a fifty percent increase in EPA following both active treatments suggesting that adherence to the treatment was very good. However, the results indicated no effect of either the multinutrient supplement or DHA-rich FO on either the NIRS or cognitive performance outcomes. 

In contrast to the current study, previous intervention trials that have assessed the effect of *n*-3 PUFAs on cerebral hemodynamics during cognitive task completion have shown modulation of cerebral hemodynamic response during tasks following treatment in both young and older adults. For example, Jackson *et al*. [43] revealed increased hemodynamic response in the prefrontal cortex following 12 weeks supplementation with 1 or 2 g/day DHA-rich fish oil (450/900 mg DHA + 90/180 mg EPA) in healthy young adults (18–35 years), with no reported effect on cognitive performance. Similarly, Konagai *et al*. [39] reported increased concentrations of oxygenated hemoglobin in the dorsolateral prefrontal cortex in healthy older adults (61–72 years) during performance of a working memory task (2-back task) following 12 weeks supplementation with 2 g/d sardine oil (251 mg DHA + 491 mg EPA) or 2 g/day krill oil (92 mg DHA + 193 mg EPA). Both of these studies showed evidence of modulation of cerebral hemodynamics in the prefrontal cortex in healthy participants by *n*-3 PUFAs after just 12 weeks supplementation that was not replicated in the present study. 

As noted above, continuous wave NIRS generates concentration change, rather than quantitative data, and therefore only provides a measure of acute changes in hemodynamics during each discrete recording session. It therefore provides no direct measure of any changes that have taken place between recording sessions. One interesting possibility is that chronic (*i.e*., longer than 12 weeks) administration with the active treatments resulted in (undetected) long-lasting changes in CBF parameters, and therefore hemodynamic response to cognitive tasks followed the same pattern as placebo. DHA in particular is known to have numerous and complex actions in the cerebro(vascular) system which may be enhanced by supplementation including facilitation of the cerebrovascular coupling mechanism [29,30], promotion of endothelial fluidity and membrane-bound protein function [44] as well as modulating the production of nitric oxide [45], a potent second messenger that regulates the local cerebral blood flow response to neural activity in the brain [46]. It is impossible to make any assertions about changes in resting CBF using data from the current study, but this could be achieved by using a quantitative NIRS system in future studies, as well as including interim assessments to track the time course of effects. 

Another consideration is the nature of the active supplements themselves, which both contained approximately the same amount of DHA (900 mg) and >200 mg EPA. Whilst the focus has been concentrated on DHA with regard to augmenting cognitive performance in older adults, it may be that EPA is more relevant to CBF parameters and possibly also cognitive function in this population. For example, in the study by Konagai *et al*. [39], described above, the concentration of EPA in the sardine oil was double that of DHA. In addition, the study reported by Witte *et al*. [12] also administered a supplement that was more highly concentrated in EPA than DHA (880 mg DHA + 1320 mg EPA), although it must be noted that the observed effects of treatment in this study may also be attributable to the large dose of DHA alone. Only two groups of researchers have assessed the effects of EPA- and DHA-rich supplements under the same experimental conditions [47,48,49,50,51] and yet these studies suggest that effect of supplemental EPA on brain function and behavior requires further exploration, and is an issue worthy of future investigation.

The second observation of the present study was that neither of the active treatments had an effect on any of the cognitive performance measures, nor participants’ rating of mental fatigue during completion of each of the CDB repetitions. A number of intervention studies have also reported no effect of *n*-3 PUFAs on cognitive function in non-demented older adults [52,53,54]. However, in all these studies the dose of *n*-3 PUFAs was much lower than in the present study; significant beneficial effects of treatment have been more frequently observed at doses >850 mg/day when administered for at least 6 months [10,11,12,13,14]. In addition, a recent pilot study that administered the same multinutrient supplement and at the same dose as the present study in older women for 6 months (*N* = 27) reported a beneficial effect of treatment on motor task performance and word recall (episodic memory) [14]. The tasks that were completed by participants in the present study were selected on the basis that they have previously been shown to activate the pre frontal cortex in previous studies that have assessed cerebral hemodynamic response to task using NIRS [34,37,38]. Therefore, it is possible that the cognitive tasks that were selected for the present study were not sensitive to the neurophysiological changes as a consequence of either *n*-3 PUFAs or the *n*-3 PUFA multinutrient supplement. Indeed, emerging evidence suggests that episodic memory tasks may be particularly sensitive to dietary supplementation with *n*-3 PUFAs [55]. However, the lack of an effect following supplementation with the *n*-3 PUFA multinutrient must be considered separately. Acute administration of 360 mg *Ginkgo biloba* resulted in improved performance on both the Serial 3 and 7 subtractions tasks in healthy young adults [56]. Therefore, lack of an effect in the present study on these tasks could indicate that this effect is not seen at lower doses, following chronic use or indeed in older, compared to younger, adults. Other trials that have administered *Ginkgo biloba* in cognitively intact older adults have yielded mixed results with some trials showing positive effects on memory performance [57,58] or no effect at all [59,60]. Similarly, whilst B vitamin deficiency in general has been linked to cognitive impairment [61], long-term (2 years) supplementation with folic acid and vitamin B_12_ did not result any benefit on performance of a broad range of cognitive tasks in healthy older adults [62]. Overall it is beginning to be recognized that a single nutrient approach to enhancing and/or preventing decline in cognitive function in aging, whilst holding great academic importance, is outdated and that the next step is to explore synergies between nutrients which could potentially yield better results [63]. For example, administration of DHA combined with PS in healthy older adults resulted in improved performance on memory and sustained attention—similar to the RVIP task in the present study—outcome measures [64,65]. Whilst the current study did not reveal any effects of the *n*-3 PUFA multinutrient supplement with regard to modulation of cerebral hemodynamic response to task, evidence from other studies suggest that *n*-3 PUFA multinutrients may be effective in patients with mild Alzheimer’s disease [32,33], or improving performance in cognitively intact individuals on tasks tapping into other cognitive domains than were assessed in the present study [14]. 

Finally, it should be noted that the sample of participants that were enrolled in the present study consumed less than one portion of oily fish per week and did not consume any *n*-3 PUFA containing supplements. However, the baseline values for capillary whole blood DHA concentrations in the present study were higher than that of fingerprick samples drawn from Scottish [41] and Italian [66] populations, and even higher than reported in an American sample that included regular consumers of *n*-3 PUFA supplements [67]. It has been suggested that individuals with the lowest level of *n*-3 PUFA intake are at the most risk for cognitive decline [68], and a recent systematic review of the effects of *n*-3 PUFA supplementation on cognitive function (across all age groups) concluded that *n*-3 PUFA supplements have a beneficial effect on short term memory in those that are deficient in *n*-3 PUFA only [69]. Therefore, despite the large increase in tissue concentrations of *n*-3 PUFAs that were observed in the current trial, the baseline fatty acid status of the sample indicates that *n*-3 PUFA intake from other sources besides oily fish may have been at least adequate, despite not being optimal, which could have contributed to the null findings. Taken from a different perspective however, the present results equally suggest that hemodynamic response to and performance on the cognitive tasks selected in the present trial are not modulated by *n*-3 PUFAs alone or combined with other nutrients in this population. 

## 5. Conclusions

The results from this study did not support the findings of a number of recent reports which have revealed a modulatory effect of *n*-3 PUFAs on cerebral hemodynamics and also those that have reported a beneficial effect of *n*-3 PUFAs and *n*-3 PUFA multinutrient supplements on cognitive function. The present study was limited by the cognitive tasks that were utilized which may not be sensitive to increased intake of *n*-3 PUFAs or any other the other ingredients contained within the multinutrient supplement in this population. Despite these limitations, it is important that the effects and underlying mechanisms of multinutrient supplements continue to be explored as an approach to supporting or even augmenting cognitive function during aging.
